# Physical fitness among urban and rural Ecuadorian adolescents and its association with blood lipids: a cross sectional study

**DOI:** 10.1186/1471-2431-14-106

**Published:** 2014-04-18

**Authors:** Susana Andrade, Angélica Ochoa-Avilés, Carl Lachat, Paulina Escobar, Roosmarijn Verstraeten, John Van Camp, Silvana Donoso, Rosendo Rojas, Greet Cardon, Patrick Kolsteren

**Affiliations:** 1 Food Nutrition and Health Program, Universidad de Cuenca, Avenida 12 de Abril s/n Ciudadela Universitaria, Cuenca, Ecuador EC010107; 2 Department of Food Safety and Food Quality, Ghent University, Coupure Links 653, 9000 Ghent, Belgium; 3 Nutrition and Child Health Unit, Department of Public Health, Prince Leopold Institute of Tropical Medicine, Nationalestraat 155, 2000 Antwerp, Belgium; 4 Department of Movement and Sports Sciences, Ghent University, Watersportlaan 2, 9000 Gent, Belgium

**Keywords:** Adolescent, Physical fitness, Urban health, Dyslipidemia, Ecuador

## Abstract

**Background:**

Physical fitness has been proposed as a marker for health during adolescence. Currently, little is known about physical fitness and its association with blood lipid profile in adolescents from low and middle-income countries. The aim of this study is therefore to assess physical fitness among urban and rural adolescents and its associations with blood lipid profile in a middle-income country.

**Methods:**

A cross-sectional study was conducted between January 2008 and April 2009 in 648 Ecuadorian adolescents (52.3% boys), aged 11 to 15 years, attending secondary schools in Cuenca (urban n = 490) and Nabón (rural n = 158). Data collection included anthropometric measures, application of the EUROFIT battery, dietary intake (2-day 24 h recall), socio-demographic characteristics, and blood samples from a subsample (n = 301). The FITNESGRAM standards were used to evaluate fitness. The associations of fitness and residential location with blood lipid profile were assessed by linear and logistic regression after adjusting for confounding factors.

**Results:**

The majority (59%) of the adolescents exhibited low levels of aerobic capacity as defined by the FITNESSGRAM standards. Urban adolescents had significantly higher mean scores in five EUROFIT tests (20 m shuttle, speed shuttle run, plate tapping, sit-up and vertical jump) and significantly most favorable improved plasma lipid profile (triglycerides and HDL) as compared to rural adolescents. There was a weak association between blood lipid profile and physical fitness in both urban and rural adolescents, even after adjustment for confounding factors.

**Conclusions:**

Physical fitness, in our sample of Ecuadorian adolescents, was generally poor. Urban adolescents had better physical fitness and blood lipid profiles than rural adolescents. The differences in fitness did not explain those in blood lipid profile between urban and rural adolescents.

## Background

Non-communicable disease, predominantly cardiovascular disease and type II diabetes, have become leading causes of death and disability, accounting for 80% of total deaths in low- and middle-income countries worldwide [1]. Current evidence indicates that the development of non-communicable disease starts early in life [2] and is associated with poor physical fitness, low physical activity levels [3] and inadequate diet [4]. Physical fitness has a closer association to the occurrence of both cardiovascular disease, and cardiovascular risk factors, than do physical activity levels [3,5]. Physical fitness, in contrast to physical activity, is stable over several months within an individual [6] and has therefore been proposed as a marker for cardiovascular risk in children and adolescents [7].

Recently, low- and middle-income countries have experienced a rapid increase in the development of risk factors for non-communicable disease among young people. Ecuador is no exception. A recent study in a group of urban and rural Ecuadorian adolescents [8] reported that dyslipidemia, abdominal obesity and overweight were prevalent in 34.2%, 19.7% and 18.0% of the population. Although elevated levels of dyslipidemia were found in both urban and rural populations, dyslipidemia was higher in the rural group. Unexpectedly, a previous analysis showed that dietary intake was weakly associated with plasma lipid (Ochoa–Aviles unpublished data). Therefore, it was hypothesized that an association of blood lipids with physical fitness is probable, and is a dimension of analysis that could further be explored.

There are few studies that have assessed physical fitness [9-13] and its association with cardiovascular risk factors in low- and middle- income countries [14]. In fact, only a single study in adolescents has investigated a comprehensive assortment of physical fitness components such as: speed, muscular endurance/strength, cardio-respiratory endurance and flexibility [11], and only one has assessed the association of cardiorespiratory fitness with dyslipidemia [14]. To the author’s knowledge no studies thus far have assessed associations of blood lipid levels with a similar variety of fitness components (speed, muscular strength endurance, cardio-respiratory endurance, flexibility and balance) according to residential location (rural vs. urban). This is surprising considering incidence of cardiovascular risk factors is known to vary along with environmental factors, such as location of residence (urban vs. rural areas) [15]. Rural areas differ considerably to urban areas, i.e. in terms of available health services, medical specialists [15], sport facilities or recreational areas [16], transportation (traffic and means of transport), safety issues [17], food availability [4] and formal education, among others [15].

This study has two objectives: i) to assess the physical fitness in a group of urban and rural Ecuadorian adolescents and ii) to analyze the associations of physical fitness and lipid profile in adolescents according to residential location.

## Methods

### Participants

Data were collected in Cuenca city and Nabón canton, which are both located in the Azuay province in the south of Ecuador at 2550 and 3300 meters above sea level, respectively. Cuenca is considered an urban area, as 60% of the 505,000 habitants are city dwellers, while Nabón is in a rural area with approximately 90% of 15,000 inhabitants living in the surrounding rural areas. Data from the National Institute of Statistics in Ecuador indicate that the estimated prevalence of poverty is substantially higher in Nabón compared to Cuenca (93% vs. 2% respectively) [18].

**Figure 1:**
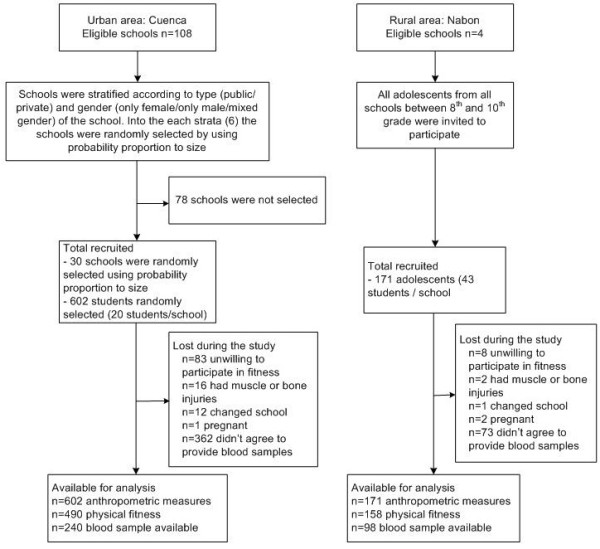
Flowchart for sample selection of study participants, Cuenca and Nabón, Ecuador 2009.

This cross-sectional study involved 773 students between the ages of 10 to 16 years old (Figure 1). A two-stage cluster sampling of schools and classes was used to select adolescents in the urban area. Schools were grouped in six strata according to (i) their classification (public or private school) and (ii) school gender (male only, female only and co-ed schools). In the first stage of sampling, 30 schools were selected with a probability proportionate to student population. In the second stage, all students between 8^th^ and 10^th^ grade were listed, and out of this sample 30 adolescents were randomly selected within each school. In the rural area, all children from 8^th^, 9^th^ and 10^th^ grade attending all four schools of Nabón were invited to participate.

Data on physical fitness were obtained from a sample of 158 and 490 in rural and urban adolescents, respectively. There were no differences in mean age (P = 0.62) or BMI (P = 0.36) between the total population and the sample of adolescents who agreed to participate in the fitness test. Power analysis showed that this sample size was sufficient to estimate the physical fitness with a precision of 11.4% and a power of 80%. A volunteering sub-sample of 301 adolescents from both the rural (n = 90) and the urban (n = 211) area provided blood samples to determine biochemical parameters.

#### Ethical approval

Ethical committees from Universidad Central in Quito-Ecuador and the Ghent University Hospital Belgium approved the protocols for anthropometry, physical fitness and biochemical determinations (Nr 125 2008/462 and 2008100–97 respectively). Adolescents (acceptance rate 85%) and their parents or guardians (participation rate 90%) provided written consent for the study. Overall, adolescents were excluded from the sampling if they had reported a concomitant chronic disease that interfered with their normal diet and physical activity, had physical disabilities or were pregnant. In the assessment of physical fitness, adolescents with chronic muscle pain or bone fractures were not able to perform any of the tests (Figure 1).

#### Outcome measurements

Prior to data collection, medical doctors, nutritionists and health professionals were trained for three full days to assess outcomes: anthropometrics, physical fitness, unsatisfied basic needs and 24 hour recall questionnaires. A manual with standardized procedures was developed for the purpose of the study and used during the training. Two biochemists were in charge of collecting and analyzing blood samples.

#### Anthropometrics

Anthropometric variables were measured in duplicate by two independently trained staff following standardized procedure [19]. The children wore light clothes, no shoes and field workers made efforts to optimize the privacy of the participants. Height was measured using a mechanical stadiometer model SECA 216 and recorded to the nearest mm. Weight was measured using a digital balance model SECA 803 and recorded to the nearest 100 g. The BMI (calculated as weight/height^2^) was used to adjust the association between blood lipid and physical fitness parameters.

#### Physical fitness

Physical fitness was measured using the EUROFIT [20] test battery, which is considered a valid and standardized test for adolescents [21]. The reliability and validity of fitness tests in adolescents has been widely documented [11,21-24]. EUROFIT is a valid method to evaluate fitness components [25], it offers advantages over other objective methods such as AAPHERD, CAHPER and Canadian as it assesses health-related fitness [25,26]. Furthermore, this test is easy to apply and can be performed in large groups, and requires few materials. A potential disadvantage of EUROFIT could be that scoring might be considered subjective, since practice and motivation levels can influence the score attained [20].

In each school the EUROFIT [20] test battery was used to assess different dimensions of physical fitness with nine tests: cardio-respiratory endurance (shuttle run 20 m measured in laps), strength (handgrip measured in kilogram-force and vertical jump measured in centimeter), muscular endurance (bent arm hang measured in seconds and sit-ups measured in the number of sit-ups/30 seconds), speed (shuttle run 10x5 m measured in seconds and plate tapping as time needed to complete 25 cycles), flexibility (sit and reach measured in centimeter) and balance (flamingo balance measured as the number of tries needed to keep balance for the duration of one minute). High scores indicate higher levels of physical fitness, apart from the shuttle run 10 × 5 m, plate tapping and flamingo balance, for which lower scores indicate a higher level of fitness. The physical fitness assessment lasted approximately two hours per school. At the end of each testing day, all forms used for data collection were taken up and revised by the supervisors. In case of missing registration forms, the researcher returned to the school to collect them. A total of 125 (16.2%) adolescents did not perform the fitness tests, most of them declined to participate (n = 91), or had otherwise experienced bone/muscle injury (n = 18) or had changed schools (n = 13) (Figure 1).

The FITNESSGRAM standards [27] for age and gender were used to classify adolescents into those who had reached the Healthy Fitness Zone, defined as the minimum level of aerobic capacity (in ml/kg/min units of VO_2max_) that provides protection against health risks associated with inadequate fitness. Aerobic capacity was determined according to the results of the aerobic capacity test (20 m shuttle run). For girls, standards values range from 40.2 ml/kg/min to 38.8 ml/kg/min across the developmental transition, 11 to 17 years old. For boys, values start around 40.2 ml/kg/min, rising to 44.2 ml/kg/min [27]. To obtain the VO_2max_ from the result of the 20 m shuttle run, the following validated equation was used VO2max = 41.77 + 0.49 (laps) - 0.0029 (laps)^2^ - 0.62 BMI + 0.35 (gender* age); where gender = 0 for girls, 1 for boys [28].

#### Unsatisfied Basic Needs (UBN)

The Integrated Social Indicator System for Ecuador was used to determine the socio-economic status per adolescent household. We adopted this method to enhance comparability of our findings with national data. The method classifies a household as “poor” when one or more deficiencies in access to education, health, nutrition, housing, urban services (electricity, potable water or waste recollection) and employment is reported. All households with one, or no deficiencies, are classified as “better off”. The unsatisfied basic needs data were used to adjust the analysis the associations of physical fitness and blood lipid parameters.

#### Energy intake

A detailed description of the dietary intake is described elsewhere (Ochoa-Aviles unpublished data). The food intake data (total energy intake in particular) were used primarily to adjust the associations of the physical fitness and blood lipid parameters. To estimate food intake two interview-administered 24 h dietary recalls were taken, the first in a weekday and second on the weekend. The procedures used to assess the dietary intake were in line with the recommendations of current literature [29]. Local utensils were selected in order to standardize food portion size. The Ecuadorian food composition database is considered outdated, and therefore was not used. Following food composition databases were used instead: U.S (USDA, 2012), Mexican (INNSZ, 2012), Central America (INCAP/OPS, 2012) and Peruvian (CENAN/INS, 2008). The data was entered in Lucille, a food intake program developed by Gent University (Gent University, http://www.foodscience.ugent.be/nutriFOODchem/foodintake, Gent, Belgium). The energy intake was analyzed using Stata version 11.0 (Stata Corporation, Texas, USA).

#### Blood lipid profile

After an overnight fast of minimum 8 hours, a blood sample of 10 ml was collected by venipuncture at the antecubital vein. The blood samples were kept on ice without anticoagulant. Subsequently, serum was separated by two centrifugations at 4000 rpm for 5 min. Serum total cholesterol (TC; CHOD-PAP kit, Human, Wiesbaden-Germany) and triglycerides (TG; GPO-PAP kit, Human, Wiesbaden-Germany) were analyzed by a calorimetric enzymatic method [30] on a Genesys 10 Thermo Scientific spectrophotometer (Madison, Wisconsin-USA). High-density lipoprotein cholesterol (HDL) was separated after sodium phosphotungstate-magnesium chloride precipitation [31]. The Friedewald formula was used to calculate low-density lipoprotein cholesterol (LDL) [32].

The intra-assay and inter-assay coefficients of variation for serum total cholesterol were 3.3% and 5.3% and for triglycerides, 5.7% and 0.9% respectively. The acceptable level was for TC < 170 mg/dl, TG < 150 mg/dl, HDL > 35 mg/dl and LDL < 110 mg/dl. The acceptable levels for TC, HDL and LDL were in accordance with guidelines of the National Cholesterol Education Program [33] for children and adolescents, while the acceptable level of TG complies with the consensus definition of metabolic syndrome in children and adolescents [34]. Adolescents were classified as having dyslipidemia when at least one of the lipid profile parameters reached risk level [35].

#### Data quality and analysis

Data were entered in duplicate into EpiData (EpiData Association, Odense, Denmark) by two independent researchers and cross-checked for errors. Any discrepancy was corrected using the original forms. Data were analyzed using Stata version 11.0 (Stata Corporation, Texas, USA). The analysis was adjusted for the cluster sampling design by using the Stata svy command and the level of significance was set at p < 0.05. Normality of data was checked using the skewness and kurtosis test. Dependent variables that were not normally distributed were log transformed before inclusion in the models. In this case, beta coefficients were back transformed and expressed as percentage differences (estimate-1*100). Prior to analysis, differences between the total sample and subsample with blood parameters were evaluated using a t-test for numerical data and chi-square test for categorical data. The characteristics of sample and outcomes of the study are presented as mean (standard deviation) by gender and location of residence (rural/urban).

Linear regression models were used for continuous outcomes to test: (i) differences in physical fitness, blood lipid profile and anthropometric variables by gender and by residential location, all of which were adjusted by BMI and gender, when appropriate, (ii) physical fitness differences among adolescents who did, or did not, reach the Healthy Fitness Zone adjusted by BMI and gender, (iii) associations between physical fitness and BMI (model: Fitness = β_0_ + β_1_ residential location + β_2_ gender + β_3_ BMI + β_4_UBN + β_5_BMI*residence + е), and (iv) associations between blood lipid level with physical fitness (model: Lipids = β_0_ + β_1_fitness + β_2_ residential location + β_3_gender + β_4_BMI + β_5_UBN + β_6_energy intake per person + β_7_fitness*residence + е). Logistic regression was used to test the association of physical fitness with dyslipidemia. The associations of physical fitness with BMI and blood lipid were stratified for residential location when interaction terms were significant (p_interaction_ < 0.1). As this study was exploratory and not confirmatory, we did not adjust for multiple testing [36]. Nevertheless, we also report our results on associations between blood lipid profiles and EUROFIT tests after applying a Bonferroni correction using an adjusted p-value of 0.005.

## Results

In this study data from 648 adolescents were analyzed (83.3% of total sample). The average age was 13.6 ± 1.2 years and 52.3% of the population was male. In the rural area, more females (61.4%; n = 97) participated (p < 0.001) than in the urban area (43.3%; n = 212). According to the result of the aerobic capacity test, 59% of the adolescents (55.0% urban and 73.5% rural) fell below the Healthly Fitness Zone. Physical fitness with respect to the other EUROFIT tests was lower among adolescents whose aerobic capacity was below the Healthy Fitness Zone, with significant differences in all tests (p < 0.05) except for the plate tapping (p = 0.12).

There was no significant difference in mean age (p = 0.54), BMI (p = 0.35), cardiopulmonary fitness (p = 0.99), speed shuttle run (p = 0.44), plate tapping (p = 0.71), sit and reach test (p = 0.54), sit-up (p = 0.30), vertical jump (p = 0.89), bent arm hang (p = 0.11), handgrip (p = 0.55) and flamingo (p = 0.09) tests between the subsample providing blood samples and the total population that participated in physical fitness assessment. Only the gender balance (p = 0.03) was marginally different between the subsample who provided blood sample and the whole sample (52.8% girls in the subsample versus 47.7% girls in the total sample).

Differences in physical fitness, anthropometric indexes and blood lipids by gender and by residence are shown in Table 1. After adjusting for BMI, boys showed higher levels of cardiorespiratory, speed, strength, endurance and balance in all EUROFIT tests compared with girls, except for the sit and reach test (p < 0.01). Blood lipid levels, however, showed no significant gender differences, with the exception of triglyceride levels (p = 0.03), which were higher in girls, after adjustment for BMI. With respect to residential location, urban adolescents had a higher mean score in the 20 m shuttle test (p = 0.01), speed shuttle run (p < 0.01), plate tapping (p < 0.01), sit-up (p < 0.01) and vertical jump (p < 0.01). In terms of blood lipid profiles, mean triglycerides (p = 0.02) and HDL (p < 0.01) revealed urban adolescents had an improved blood lipid profiles as compared to rural adolescents. Therefore, the proportion of the population with dyslipidemia was significantly lower in the urban area than in the rural area (28.9% vs. 46.7%, P < 0.01).

**Table 1:** Anthropometry, physical fitness and blood lipids of Ecuadorian adolescents stratified by gender and by residential location

	**Boys**		**Girls**		**P**^**a**^	**Urban**		**Rural**		**P**^**b**^
	**n**	**Mean (SD)**	**n**	**Mean (SD)**		**n**	**Mean (SD)**	**n**	**Mean (SD)**	
Age	334	13.6 (1.2)	306	13.6 (1.2)	0.36	487	13.7 (1.1)	153	13.5 (1.5)	0.48
Body mass index (kg/m^2)	334	19.9 (3.1)	306	20.5 (3.0)	0.02	482	20.3 (3.1)	158	20.0 (2.9)	0.39
Weight (kg)	336	45.9 (10.3)	307	45.3 (8.3)	0.76	485	46.7 (9.5)	158	42.3 (8.3)	<0.01
Height (cm)	336	151.6 (10.3)	307	149.1 (6.9)	<0.01	485	151.9 (8.7)	158	145.9 (7.9)	<0.01
**Physical fitness**										
*Cardiopulmonary fitness*										
20 m shuttle test (laps)	313	3.6 (1.4)	285	2.8 (0.9)	<0.01	442	3.4 (1.3)	156	2.7 (0.9)	0.01
20 m shuttle test (ml/kg/min)	303	43.0 (5.0)	279	35.4 (3.9)	<0.01	431	40.2 (6.0)	151	37.1 (5.0)	0.02
FITNESSGRAM (% who are on the Healthy Fitness Zone)	303	63.4 (48.3)	279	15.1 (35.8)	<0.01	431	45.0 (49.8)	151	26.5 (44.3)	0.19
*Speed-agility*										
Speed shuttle run (s)	338	23.3(2.0)	309	26.6 (2.7)	<0.01	489	24.4 (2.6)	158	26.3 (3.2)	<0.01
Plate tapping (s)	339	14.6 (2.1)	309	17.0 (2.5)	<0.01	490	15.3 (2.2)	158	17.2 (3.0)	<0.01
*Flexibility*										
Sit and reach (cm)	338	19.0 (6.6)	309	20.4 (7.0)	<0.01	489	19.4 (6.8)	158	20.5 (6.7)	0.52
*Muscle strength and endurance*										
Sit-up (number/30 s)	337	16.1 (3.7)	308	11.4 (3.9)	<0.01	488	14.7 (4.2)	157	11.4 (4.3)	<0.01
Vertical jump (cm)	337	29.1 (6.8)	308	23.6 (5.7)	<0.01	487	27.9 (6.5)	158	22.4 (6.2)	<0.01
Bent arm hang (s)	332	10.0 (9.1)	308	3.2 (3.0)	<0.01	483	7.3 (7.8)	157	7.1 (8.4)	0.21
Handgrip (kgf)	338	24.7 (8.0)	309	20.4 (4.8)	<0.01	489	23.2 (7.1)	158	21.2 (6.3)	0.35
*Balance*										
Flamingo (trying/1 min)	322	13.8 (5.4)	285	15.4 (5.2)	0.02	464	14.5 (5.3)	143	14.7 (5.5)	0.99
**Blood lipid profile**										
Cholesterol (mg/dL)	142	144.8 (32.7)	159	147.8 (31.7)	0.65	211	144.5 (31.3)	90	159.7 (33.8)	0.53
HDL (mg/dL)	142	51.1 (12.8)	159	48.6 (11.5)	0.24	211	51.1 (11.9)	90	46.6 (12.4)	<0.01
LDL (mg/dL)	142	75.3 (30.9)	159	78.4 (26.8)	0.66	211	74.7 (28.1)	90	82.1 (30.0)	0.42
Triglyceride	142	91.9 (48.0)	159	104.2 (58.4)	0.04	211	93.6 (54.3)	90	109.5 (52.0)	0.02

^a^p-value adjusted for BMI and clustering, ^b^p-value adjusted for gender and clustering.

The associations between fitness and BMI are shown in Table 2. The interaction in terms of BMI-residence was significant for speed shuttle run, plate tapping, sit up, vertical jump, bent arm hang and the proportion adolescents who reached the Healthy Fitness Zone. In the total sample, BMI was significantly associated with low performance on the 20 m shuttle test and flamingo, and with high performance on hang grip (p < 0.01 for all tests). When the associations between the fitness tests and BMI were analyzed according to residential location, the results showed that the proportion of adolescents that reach the Healthy Fitness Zone in both urban and rural areas decreased significantly as mean BMI increased. In addition, in both rural and urban areas the improved scores the performance on the speed shuttle run and longer duration of bent arm hang were significant, and inversely associated with BMI. In both areas, the associations between BMI with plate tapping and vertical jump test were not significant. The only difference, when considering residential location, was the association between the sit up test and BMI which was only significant in urban adolescents.

**Table 2:** Association between physical fitness and BMI of Ecuadorian adolescents stratified by residential location

**Physical fitness**	**Interaction BMI & residence**	**All**		**Urban**		**Rural**	
	**p**^**a**^	**Β%**	**p**^**a**^	**Β%**	**p**^**a**^	**Β%**	**p**^**a**^
*Cardiopulmonary fitness*							
20 m shuttle test (laps)	0.24	-2.39	<0.01	-	-	-	-
FITNESSGRAM (% who are on the Healthy Fitness Zone)	0.02	-	-	-6.49	<0.01	-3.82	0.03
*Speed-agility*							
Speed shuttle run (s)	<0.01^b^	-	-	0.47	<0.01	0.92	<0.01
Plate tapping (s)	<0.01^b^	-	-	-0.13	0.52	0.90	0.11
*Flexibility*							
Sit and reach (cm)	0.63	-0.14	0.77	-	-	-	-
*Muscle strength and endurance*							
Sit-up (number/30 s)	0.09^b^	-	-	-1.60	<0.01	-2.50	0.20
Vertical jump (cm)	0.06^b^	-	-	-0.70	0.11	1.37	0.31
Bent arm hang (s)	0.06^b^	-	-	-11.70	<0.01	-10.45	0.01
Handgrip (kgf)	0.13	3.67	<0.01	-	-	-	-
*Balance*							
Flamingo (trying/1 min)	0.58	2.26	<0.01	-	-	-	-

^a^Analysis adjusted for gender, socio economics status and cluster design, ^b^Significant interactions.

The interaction terms of residence x physical fitness were highly significant for cholesterol and LDL. The interaction term for cholesterol was significant with five EUROFIT tests, while for LDL, interaction terms were significant with four EUROFIT tests. In addition, the association between cholesterol/LDL with the proportion of adolescents who reached the Healthy Fitness Zone was significantly different between urban and rural adolescents (Table 3).

**Table 3:** Significance of physical fitnessXresidence interaction terms in relation to blood lipid profile in Ecuadorian adolescents, Cuenca- Nabón, Ecuador, 2009

	**Interaction fitness X residence**^**a**^				
**Physical fitness**	**Dyslipidemia**	**Cholesterol**	**HDL**	**LDL**	**Triglyceride**
*Cardiopulmonary fitness*					
20 m shuttle test (laps)	0.30	0.17	0.17	0.15	0.27
FITNESSGRAM (% who are on the Healthy Fitness Zone)	0.51	**<0.01**	0.17	**<0.01**	0.95
*Speed-agility*					
Speed shuttle run (s)	0.52	**0.08**	0.50	**0.05**	0.27
Plate tapping (s)	**0.09**	0.20	**0.06**	0.14	0.86
*Flexibility*					
Sit and reach (cm)	0.80	**0.01**	0.47	**<0.01**	0.69
*Muscle strength and endurance*					
Sit-up (number/30 s)	0.24	**<0.01**	0.68	**<0.01**	0.41
Vertical jump (cm)	0.99	**0.10**	0.79	0.18	0.58
Bent arm hang (s)	0.54	**0.03**	0.86	**<0.01**	0.22
Handgrip (kgf)	**0.06**	0.72	0.22	0.23	**0.02**
*Balance*					
Flamingo (trying/1 min)	0.99	0.64	0.26	0.32	0.22

^a^Analysis were adjusted for gender, BMI, socio economics status, energy intake per day and cluster design.

Significant level set to p ≤ 0.10.

The associations between the physical fitness tests and blood lipid profile were weak (Table 4). Overall, dyslipidemia was negatively related to performance in bent arm hang. There were also significant associations between the plate-taping test with HDL and triglycerides. As time increased in seconds for the EUROFIT test, HDL decreased and triglycerides increased. In the urban area there was an inverse association of bent-arm-hang and handgrip with cholesterol and LDL. In the rural area, adolescents who reached the Healthy Fitness Zone according to the FITNESSGRAM standards had significantly lower cholesterol and LDL levels. Although, after the Bonferroni correction only the association between cholesterol levels and the adolescents who reached the Healthy Fitness Zone according to the FITNESSGRAM standards remained significant.

**Table 4:** Associations of physical fitness on blood lipids in Ecuadorian adolescents, Cuenca-Nabon, Ecuador, 2009

	**Dyslipidemia**		**Cholesterol**				**HDL**		**LDL**				**Triglyceride**	
**Test eurofit**			**Urban**		**Rural**				**Urban**		**Rural**			
*Cardiopulmonary fitness*	β	p^a^	β%	p^b^	β%	p^b^	β%	p^a^	β%	p^b^	β%	p^b^	β%	p^a^
20 m shuttle test (laps)	0.85	0.20	-1.54	0.18	-2.70	0.11	-0.53	0.65	-1.72	0.34	-5.70	0.13	-0.23	0.95
FITNESSGRAM (% who are on the Healthy Fitness Zone)	0.85	0.46	-5.25	0.14	-8.91	**<0.01**	-5.31	0.08	-3.31	0.46	-11.71	**0.04**	-4.03	0.67
*Speed-agility*														
Speed shuttle run (s)	1.00	0.48	0.07	0.32	0.10	0.27	0.003	0.97	0.06	0.60	0.23	0.32	0.15	0.29
Plate tapping (s)	0.99	0.99	0.04	0.60	-0.03	0.77	-0.10	**0.05**	0.06	0.62	-0.05	0.75	0.31	**<0.01**
*Flexibility*														
Sit and reach (cm)	1.00	0.94	-0.13	0.47	-0.81	0.08	-0.25	0.26	-0.10	0.72	-1.48	0.11	0.64	0.13
*Muscle strength and endurance*														
Sit-up (number/30 s)	0.97	0.34	-0.17	0.60	-0.66	0.14	-0.17	0.69	-0.22	0.71	-1.30	**0.06**	0.04	0.96
Vertical jump (cm)	1.00	0.57	-0.28	0.34	-0.21	0.23	0.00	0.99	-0.50	0.25	-0.25	0.70	-0.22	0.56
Bent arm hang (s)	0.99	**0.05**	-0.05	**0.03**	-0.04	0.60	0.00	0.60	-0.06	**0.04**	-0.06	0.58	0.00	0.97
Handgrip (kgf)	0.99	0.92	-0.64	**0.02**	-0.34	0.27	-0.28	0.19	-0.75	**0.05**	-0.84	0.09	-0.01	0.97
*Balance*														
Flamingo (trying/1 min)	0.99	0.63	-0.13	0.64	-0.51	0.34	0.17	0.43	-0.08	0.83	-1.19	0.17	-0.01	0.97

Results were stratified by location when the interaction term was significant (P < 0.1).

^a^p-value adjusted for gender, BMI, socio economic status, energy intake per person, residential location and clustering.

^b^p-value adjusted for gender, BMI, socio economic status, energy intake per person and cluster design.

Significant level set to p ≤ 0.05.

## Discussion

To our knowledge, this is the first study in a middle-income country that estimates physical fitness in urban and rural adolescents and explores its associations with blood lipid profiles. The findings show that more than half of the sample exhibits unhealthy levels of physical fitness. Furthermore, adolescents who had a low aerobic capacity as defined by the FITNESSGRAM had lower scores for physical tests, such as speed-agility, flexibility, muscle strength-endurance and balance. Our findings also show that urban adolescents were fitter than rural adolescents for five of the fitness test. Nevertheless, these differences in physical fitness did not explain those in lipid profile between urban and rural adolescents.

Two out of three Ecuadorian adolescents in our sample had early cardiovascular risk, defined by low aerobic capacity (20 m shuttle run). This proportion was higher than the proportion reported in Spanish [37] and Portuguese [38] adolescents. Furthermore, the group of adolescents who had a lower aerobic capacity also showed lower scores for other physical fitness components such as muscle strength and endurance. Previous research indicates that such low fitness levels can linger on into adulthood [39] where low cardiorespiratory fitness [40] or low muscular strength [41] is associated with increased mortality risk.

In general, the absolute physical fitness of our population was worse than estimates in the majority of previous studies. Adolescents from our sample had a lower cardiopulmonary performance (3.2 ± 1.3 laps) compared with Spanish [42] (6.1 ± 2.0 laps) and Belgian [43] (6.3 ± 2.3 laps) adolescents. The estimates from the speed agility components of the physical test (speed shuttle run 10 × 5 m, plate taping) were also lower compared with Spanish [42], Greek [44], Polish [45] and Belgian [43] adolescents [42-45]. The sit and reach scores were lower than those from Mexico [11], Spain [42], Poland [45] or Belgium [43]. However, the large variation between studies, when considering the results from muscle strength and endurance tests (sit-ups, vertical jump, bent-arm hang and handgrip), renders comparison to the present study difficult. For sit-ups we obtained lower absolute values compared to estimates from Spain [42], Poland [45], Turkey [10] or Belgium [43]. Also, the estimates from the handgrip test were lower than those from previous studies [10,11,42,44,45]. Conversely, for the sit and reach test, we obtained a higher score compared with Greek [44] and Turkish [10] adolescents. In our results for sit-ups our adolescents averaged higher scores than adolescents in a Mexican study [11]. The favorable fitness scores in European as compared to Ecuadorian adolescents may be a reflection of the favorable environmental conditions for physical activity found in Europe [46], as well as a longer tradition of health promotion programs [47], and genetic factors [48,49]. This hypothesis may be reinforced by the fact that our results were similar when compared to those from Mexican [11] and Colombian [13] studies, which have similar environmental and genetic patterns to those of Ecuador [48].

Compared with rural adolescents, the urban participants in our sample had a significantly better performance for the cardiopulmonary, speed-agility, and muscle strength and endurance components of the fitness test. Although these findings are in line with measurements in Mexican [11] and Polish [45] adolescents, most literature consists of contradictory results with regard to comparison of performance between urban and rural adolescents [10,50-52]. Therefore, explaining the difference between urban and rural adolescents remains speculative. Firstly, the urban adolescents in our sample were taller and heavier than rural adolescents. It has been reported that the physical fitness is influenced by body size. Taller and heavier (not necessarily overweight or obese) children may therefore have an advantage on strength, speed, power and endurance components [53]. Secondly, urbanization and better social conditions in urban areas may mean that urban adolescents have increased access to sport facilities compared to rural adolescents [54-56]. Organized sports facilities are more common in urban areas and might result in higher levels of cardio-respiratory and muscular fitness in urban adolescents [42]. Thirdly, we observed that urban schools had specialized physical education teachers in their physical education programs, while these kinds of specialized teachers were virtually absent in rural areas. In addition, a lower availability of sport facilities in rural schools might result in a lower variety of sport activities. The latter was confirmed during our observations in the schools themselves. As a point of potential bias, urban adolescents are possibly more familiar with physical fitness tests than rural adolescents [11,44]. Fourthly, chronic undernutrition during childhood instigates mechanisms of adaptation such as growth stunting and reduced muscle mass. The latter are potentially related to the physical fitness impairment during adolescence and adulthood [25]. Indeed, chronic undernutrition mainly affects children in rural areas in Ecuador [18].

To our knowledge, only a few studies have analyzed the association of blood lipid profile with multiple components of physical fitness. These studies have reported that increased cardiorespiratory fitness and muscular strength are associated with favorable lipid profiles in adolescence [7,24,38,57]. These associations were partially confirmed in our study. Total cholesterol and triglycerides were negatively associated with muscular strength in the urban area, whilst in the rural population these lipids were negatively associated with cardiorespiratory fitness.

We report that differences in blood lipid profile among urban and rural adolescents are not explained by differences in physical fitness, even after adjusting for BMI and total energy intake. The association found in this study between blood lipids and fitness was adjusted for BMI and total energy intake, as these factors have previously been found associated with blood lipids [4,7]. Mean energy intake was not significantly different (P = 0.08) between urban (1863 ± 181 kcal/day) and rural (1766 ± 153 kcal/day) adolescents. (Ochoa-Avilés unpublished data). In our sample, the relationship of different blood lipid parameters with each of the EUROFIT tests according to residential location was generally weak and non-significant.

Another possible explanation for the differences in blood lipid profile among urban and rural adolescents may be the differences in moderate to vigorous physical activity [58], or body fat distribution [59]. Physical activity and fitness have been found independently associated with certain blood lipid levels among children and adolescents [6]. For example, the favorable TG and HDL levels are inversely associated with moderate to vigorous physical activity, independent of time spent sedentary [58] and fitness [6]. In our sample, the time spent on moderate to vigorous physical activity could be longer in urban adolescents compared to rural adolescents because of differences in the availability of sport facilities and organized group sports, detailed earlier in this discussion. In addition, qualitative research performed in adolescents from Cuenca and Nabón has shown that rural adolescents felt an inability to perform physical activity in contrast to the urban adolescents (Van Royen unpublished data). This fact could lead to differences in physical activity levels between urban and rural adolescents, as self-efficacy is an important determinant of physical activity in adolescence [60]. On the other hand, total cholesterol, LDL, HDL and TG also have been associated with fat distribution measured by skin-fold thickness. Lean adolescents, as determined using the skin-fold system, have been found to have healthier blood lipid profiles compared to their heavier peers [61]. However, skin-fold thickness was not a parameter measured in the present study.

There are a few limitations of this study. Firstly, its cross-sectional nature of only allows us to establish associations and not causality. Secondly, we did not measure important variables associated with blood lipids such as physical activity, pubertal stage, sex hormone level, skin-fold thickness and familial health background. Third, the blood lipid determinations were conducted only in a subsample. Nevertheless, there were no differences in physical fitness and BMI between the subsample and the total sample. Fourth, reliability and validity of EUROIFIT were not done in our sample. Although, EUROFIT has shown good validity in previous studies performed in the region [11]. We followed the EUROFIT guidelines in order to avoid source of bias, such as learning effect, or low motivation of adolescents to do their best performance during each test [20]. Measurements of the 20 m shuttle run could be influenced by the temperature and weather conditions during the test. In Cuenca and Nabón, however, the average temperature and altitude are similar. In addition, the estimation of VO_2max_ from the FITNESSGRAM standards of the 20 m shuttle run is known to vary with the equation used. A previous study [28] has tested the degree of agreement between various equations used to estimate VO_2max_ and the actual the VO_2max_. In the present study, we used the equation that shows the highest agreement. Finally, our results could be compared with only one other similar study in a low- and middle- income country, which hinders comparison of our findings with previous data in similar populations. The trial included adolescents from high altitude urban and rural areas of Ecuador that are characterized by mixed *mestizo* (in urban area) and *Amerindian* (in rural area) ethnicities [49]. The external validity of our findings is hence limited to urban and rural schools in the regions that share these characteristics [62].

## Conclusions

The results from our study suggest that 59% of Ecuadorian adolescents have poor physical fitness. Even though urban participants showed better scores in the majority of EUROFIT tests, physical fitness of the total population was lower compared to that of adolescents from other countries. These findings call for specific health promotion programs aimed to improve physical fitness among Ecuadorian adolescents. Differences in fitness did not explain differences in blood lipid profile between urban and rural adolescents. We only found a weak association between physical fitness and blood lipid profile, even after adjustment for energy intake. Additional studies are needed to clarify the frequent occurrence of unfavorable blood lipid profiles among rural participants. Such studies might explore associations with physical activity levels, body fat distribution, risk factors at early ages, familial hypercholesterolemia and ethnic differences.

## Abbreviations

LMICs: Low- and middle- income countries; BMI: Body mass index; EUROFIT: European tests of physical fitness; VO2max: Maximal oxygen uptake; TC: Total cholesterol; TG: Triglycerides; HDL: High-density lipoprotein cholesterol; LDL: Low density lipoprotein cholesterol; UBN: Unsatisfied basic needs.

## Competing interests

The authors declare that they have no competing of interests.

## Authors’ contributions

AS and OA designed the study, coordinated and participated in its implementation, performed the analysis and interpretation of results, drafted the article, and approved the version to be published. LC and KP designed the study, performed the analysis and interpretation of results, contributed with important intellectual improvements of the article, reviewed the article and approved the final version. EP and VR designed the study, participated on implementation and quality assurance, contributed with important intellectual improvements of the article, reviewed the article and approved the final version. VJ, DS, RR and CG contributed to the interpretation of results, contributed with important intellectual improvements of the article, reviewed the article and approved the final version.

## Pre-publication history

The pre-publication history for this paper can be accessed here:

http://www.biomedcentral.com/1471-2431/14/106/prepub
